# Local-feature and global-dependency based tool wear prediction using deep learning

**DOI:** 10.1038/s41598-022-18235-3

**Published:** 2022-08-26

**Authors:** Changsen Yang, Jingtao Zhou, Enming Li, Mingwei Wang, Ting Jin

**Affiliations:** 1 School of Mechanical Engineering, Northwestern Polytechnical University, Xi’an, 710072 China; 2 Science and Technology Information Department, Shaanxi Diesel Heavy Industry Co. Ltd, Xi’an, 713105 China

**Keywords:** Mechanical engineering, Computer science

## Abstract

Evaluation of tool wear is vital in manufacturing system, since early detections on worn-out condition can ensure workpiece quality, improve machining efficiency. With the development of intelligent manufacturing, tool wear prediction technology plays an increasingly important role. However, traditional tool wear prediction methods rely on experience and knowledge of experts and are labor-extensive. Deep learning provides an effective way to extract features of raw data and establish the mapping relationship between features and targets automatically. In this paper, a new local-feature and global-dependency based tool wear prediction method is proposed. It is a hybrid approach combining manual features with automatic features. Firstly, an enhanced CNN network is designed and applied on the transformed wavelet scalogram to learn the local single-scale specific features and multi-scale correlation features automatically. Secondly, sequence of local feature vectors combining manual features with automatic features are fed into multi-layer LSTM step by step for the global dependency. A fully connected layer is then trained to predict tool wear. Finally, two statistics are proposed to illustrate the overall prediction performance and generalization ability of the model. An experiment illustrates the effectiveness of our proposed method under multiple working conditions.

## Introduction

The rise of multi-variety, variable batch and customized production mode requires the production system to be equipped with stronger monitoring ability of the production process in the complex and changeable production environment^1,2^. Production system endued with self-perception can response to the changeable production environment in time. Under the new production mode, tool wear prediction system, as an indispensable part of automatic and intelligent processing, has been paid more and more attention by researchers and engineers. Accurate prediction of tool wear during machining can be of great significance for ensuring workpiece quality, improving machining efficiency, and promoting automatic and intelligent machining^3^.

In recent years, the development of computer technology, sensor technology and signal processing technology make it possible to monitor the signal of the machining process in real time, which promotes the development of data-driven method in tool wear prediction^4,5^. Scholars have done a lot of research on data-driven tool wear prediction. The main idea of the data-driven method is to train the tool wear model based on the historical data, and then input the data collected online into the trained model to predict the tool wear. The data-driven method can be divided into traditional method and deep learning-based method.

### Traditional data-driven tool wear prediction method

Traditional data-driven tool wear modeling methods, such as fuzzy clustering^6^, support vector machine^7^, decision tree^8^ and neural network^9^, do not require in-depth analysis of the complex tool failure mechanism in the cutting process, but are a kind of methods to predict tool wear by mining correlations between wear characteristics and wear value. With the continuous development of various supporting technologies, their reliability and stability have also made great progress, and these methods have become the mainstream in tool wear prediction.

Li et al.^6^ extracted signal features of cutting vibration by applying frequency-band energy decomposition using wavelet packets. Then tool wear states can be recognized by affinities between the known state and unknown state obtained through the fuzzy clustering. Zhang et al.^10^ solved the highly nonlinear and noisy black-box modelling problems by studying the least square support vector machine (LS-SVM), and established the tool wear model of ball-end milling cutter based on LS-SVM considering the joint effect of machining conditions. Krishnakumar et al.^11^ used Dimensionality Reduction Technique to select a set of prominent features. Then classifications of tool conditions were carried out using J48 decision tree algorithm and artificial neural network (ANN). In addition, some researchers integrate physical information into the data-driven model to optimize it. Li et al.^12^ proposed a physics-informed meta-learning framework for tool wear prediction under varying wear rates. An experimental was performed to validate the effectiveness of the method.

Traditional data-driven method mentioned above requires manual extraction of tool wear features or very professional domain knowledge to build the model, and the performance of the model largely depends on the quality of the extracted features and the structure of the model. It relies heavily on the expertise of experts in related fields, which makes it less adaptable in different fields and scenes.

### Deep learning based tool wear prediction method

In recent years, thanks to the development of big-data in all walks of life and the improvement of computing power, deep learning has become a popular technology in machine learning and data-driven algorithms. With its powerful feature extraction, feature fusion and abstraction ability^13–15^, it can directly extract features from original data without relying on human experience, which makes it widely used in various industries and gradually applied in tool wear prediction^16–18^. Zhao et al.^16^ convolved and pooled the original data along the timing sequence with 1D convolutional neural network (CNN) to obtain the compressed timing features and input them into the bi-directional long short-term memory network (LSTM) to predict tool wear. Chan et al.^19^ proposed Holistic–Local Long Short-Term Memory (HLLSTM), which uses CNN to extract features from original data and uses the extracted results as input of LSTM to predict tool wear. However, when original data is directly input into the network, the model is usually overfitted due to too a large number of parameters^5^ and may not work as well due to unclear features. Therefore, some scholars combine feature engineering with deep learning to extract more abstract features from manual features^20^. Some good results have been achieved. Martinez et al.^21^ converted the time series into images using GAF imaging technique, and then input the images into CNN for training prediction and tool wear classification. Marani et al.^22^ extracted statistical features from the raw data and proposed a prediction model for tool flank wear derived from long short-term memory (LSTM) modelling, the results show that the best LSTM model demonstrates its capability to capture tool flank wear. Zhao et al.^5^ proposed local feature-based gated recurrent unit (LFGRU) networks, which combined handcrafted features design with automatic feature learning for machine health monitoring and achieved a favorable effect on tool wear prediction. Zhang et al.^23^ converted the original vibration signals into corresponding energy spectrum using wavelet packets transform, and built a tool wear monitoring model using a deep convolutional neural network (DCNN) to realize the adaptive extraction of tool wear features and the classification of wear degree. Duan et al.^24^ introduced a multi-frequency-band feature extraction structure based on a DCNN structure for sensitive feature extraction of wavelet coefficients in different frequency bands.

In addition to the methods described above, researchers are also trying to implement feature engineering using deep learning techniques. The attention mechanism^25^ is regarded as a secondary screening method of data information and is also used to improve the feature learning ability of the model. Muneer et al.^26^ proposed the LSTM model with attention mechanism to emphasize the most critical pieces of information to improve the feature learning ability of the model. The experimental results showed that the method achieved accurate remaining useful life prediction of the turbofan engine. Xu et al.^27^ introduced the channel attention mechanism into the deep learning model based on CNN and considered the weight of different feature map to enhance the performance of the model. Achievements have been made in the research above. However, there are still some potential concerns that need to be considered in the prediction of tool wear under multiple working conditions based on time series data.

1. *Data representation and feature extraction* The machinery data collected during machining process is non-linear. The representation of data is related to the quality of feature extraction and ultimately affects the effectiveness of the model^28^. How to effectively represent tool wear time-series data and extract features appropriately are still issues that need to be paid attention to.
2. *Temporal information* The machinery data collected during machining process are in a sequential form, which has the problem of being non-linear and time-variant. The transient signatures^29^ and long-term dependence relation^30^ in time-series signals are very important in indicating dynamic process relevant to tool wear and breakage. Mining the local details and the global information hidden in the time-series data, such as mutative symptom within a short period of time, or the inherent trend and correlation hidden in the whole time series, is very important for tool wear prediction.
3. *Model evaluation* Whether the prediction model is suitable in a scene depends on the reasonable evaluation of the prediction ability and stability of the model. How to evaluate prediction ability and stability of the model is a problem that needs to be considered.

In this paper, we propose a new deep-learning based tool wear prediction framework named Local-Feature and Global-Dependency based Tool Wear Prediction (LFGD-TWP). Firstly, discrete wavelet transform is used to transform the pre-processed original signals so that the wear characteristics can be manifest without loss of original signal information^28^. Then, a local feature extraction model is proposed based on the translation invariance of convolutional neural networks and its powerful non-linear feature extraction capability. The obtained wavelet scalogram is fed into convolution neural network for single-scale and multi-scale local feature extraction. Finally, local hybrid feature vectors are fed into LSTM step by step to realize tool wear prediction based on the capability of long-term dependency mining. The contributions of this paper can be summarized as follows.

1. A new hybrid tool wear prediction method combining manual features and automatic features is proposed, which is suitable for multi-sensor fusion scenarios. Therefore, the model not only inherits the powerful feature extraction capability of deep learning, but also can use artificial features to optimize the model without significantly increasing the size of the model, and the required professional knowledge does not need to reach an expert level.
2. Wavelet transform is used to manifest characteristics without losing information of original data. The sensitive features of tool wear can be further extracted and selected from wavelet coefficients, which makes it more conducive to data mining.
3. A local time series feature extraction model based on enhanced multilevel-CNN is proposed for local information in a short, relatively stable cutting period, which can automatically extract single-scale specific features and multi-scale correlation features. The global dependency mining is realized by multi-layer LSTM. Therefore, the local information and long-term dependencies hidden in the original data are taken into consideration, which can solve the problem of non-linear and time-variant in tool wear signals.
4. Two statistics are proposed to evaluate the overall prediction performance and generalization ability of the model, and the effectiveness of the model is verified.

This paper is organized as follows. In “Introduction” section, Current situations and problems of tool wear prediction are discussed and analyzed, and the research contents and results of this paper are introduced. “Local-feature and global-dependency based tool wear prediction” section presents the methodologies, including local time series data conversion, local time series feature extraction and global time series dependency mining. “Case study and experimental results” section presents the results of proposed approach on tool wear data set. “Conclusions” section presents the conclusions of this paper and future research works.

## Local-feature and global-dependency based tool wear prediction

**Figure 1:**
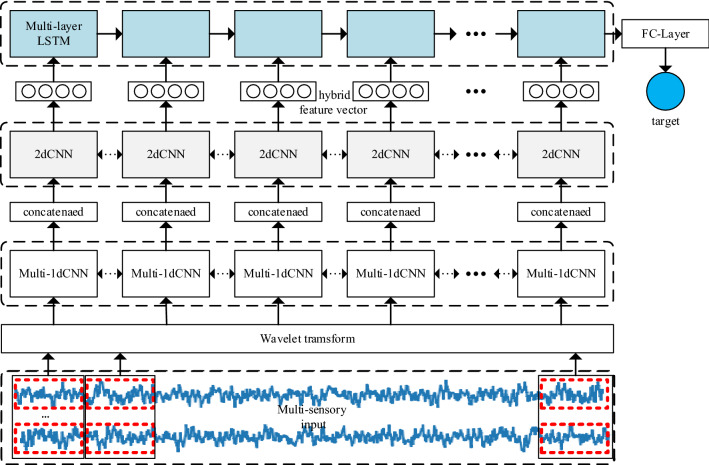
Local-feature and global-dependency based tool wear prediction.

In this section, our proposed LFGD-TWP framework will be presented in the scenario of multisensory machine monitoring. As shown in Figure 1, the enhanced CNNs are applied on the sequence of wavelet scalograms transformed from original multi-sensor data to learn local representation of wear condition. The enhanced LSTM network is applied on the sequence of local feature vectors to learn global-dependency and predict the tool wear. The input of our framework is the time series data collected during machining, which is denoted as $X = \left[ {x_{1} ,x_{2} ,...,x_{T} } \right]$ where $T$ is the length of data sample.

### Local time series data conversion

In traditional data-driven tool wear prediction method, the original data or manual features are input into the training model, which usually leads to overfitting problems or information loss. In this paper, a wavelet-based data representation method is proposed, through which the time-domain raw data are converted to images. And then we can extract tool wear features automatically using CNN automatically.

**Figure 2:**
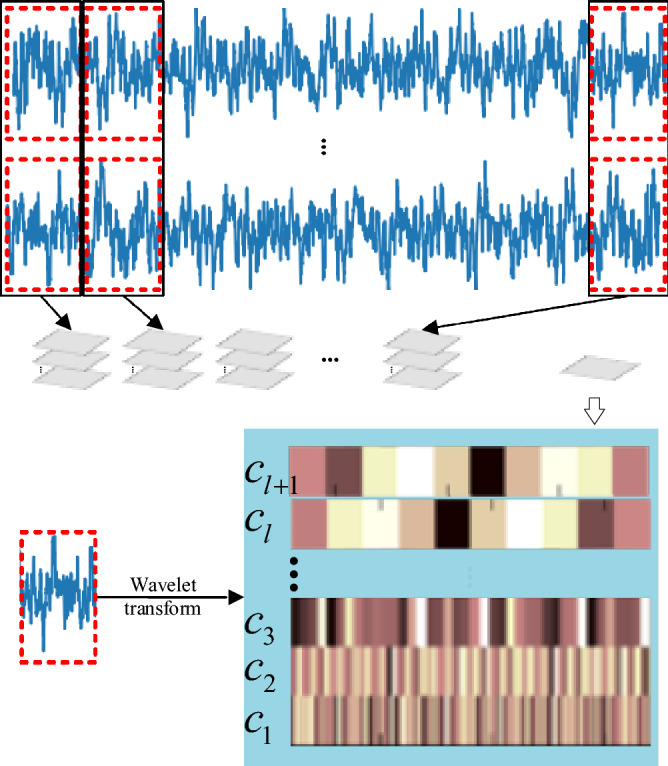
Local time series data conversion.

As shown in Fig. 2, each set of time-series raw data is first divided into N local segments. Each segment is a window of raw data with a length of $\frac{T}{N}$. Therefore, the time-series raw data can be transformed to a sequence of local segments as ${\text{X}} = \left[ {X_{1} ,X_{2} ,...,X_{N} } \right]$.Then, each local segment signal is processed by wavelet transform into a multi-scale spectrogram image to manifest the wear characteristics. The result $wt(s,\tau )$ of wavelet transform is obtained by multiplying a family of wavelets $\psi_{\tau ,s}$ with the raw data $x(t)$ along time. It decomposes the raw signal onto a time-scale plane, which each scale ‘$s$’ corresponding to specific frequency information of the original signal. The formulas of wavelet transform are as follows:

1 $$ \psi_{\tau ,s} = \left| s \right|^{ - 1/2} \psi \left( {\frac{t - \tau }{s}} \right), $$

2 $$ wt(s,\tau ) = \left| s \right|^{ - 1/2} \int\limits_{ - \infty }^{ + \infty } {x(t)\overline{\psi } } \left( {\frac{t - \tau }{s}} \right)dt, $$

3 $$ \left\langle {x(t),x(t)} \right\rangle = \int\limits_{ - \infty }^{ + \infty } {\left| {x(t)} \right|^{2} dt = \frac{1}{{C_{\psi } }}\int\limits_{ - \infty }^{ + \infty } {s^{ - 2} \int\limits_{ - \infty }^{ + \infty } {\left| {wt(s,\tau )} \right|^{2} dsd\tau } } } , $$

where $\psi (t)$ is the base wavelet and $\psi_{\tau ,s}$ is the wavelet function obtained from shifting ($\tau$) and scaling ($s$) of the base wavelet. The wavelet transformation of a signal $x(t)$ with finite energy can be performed through convolution of $x(t)$ with the complex conjugate of a family of wavelets $\psi_{\tau ,s}$ according to (2). Therefore, we get the result of wavelet transform in terms of $wt(s,\tau )$.

Discrete wavelet transform is adopted in order to avoid information redundancy and reduce computation. After the wavelet transform, each segment is represented as $ws = [c_{1} ,c_{2} ,...,c_{l + 1} ]$.Where $l$ denotes the level of decomposition. The last element $c_{l + 1}$ is approximation coefficients array and the previous elements $c_{1} ,c_{2} ...,c_{l}$ are absolute values arrays of details coefficients. From the definition of the wavelet transform and (3), it can be deduced that there is no loss of information or energy thorough the transform. Therefore, the irregular array $ws$ which is composed of $c_{1} ,c_{2} ,...,c_{l + 1}$ is another multi-scale representation of the original data.

### Local time series feature extraction using CNN

This section presents the proposed multilevel-CNN-based feature extraction method. First, multilevel 1D-CNNs are adopted to extract tool wear features from single-scale information, and then single-scale features are recombined for the sake of data balancing and multi-scale information. Finally, 2D-CNN is adopted to extract the multi-scale correlation features from the previous combined features.

#### Single-scale feature extraction and recombination

Once the raw signals have been converted to multi-scale vectors, multilevel 1D-CNNs can be trained to extract single-scale features. Convolutional layers slide the filters over input vectors to generate feature maps. It is assumed that $k_{i,j}$ filters with a window size of $m_{i,j}$ are used corresponding to the j-th convolution operation of input vector $c_{i}$. Then, the max pooling is adopted to compress generated feature maps to produce significant features. Therefore, the operation in the j-th convolution layer of the input vector $c_{i}$ can be expressed as:

4 $$ o_{i,j} : \, Conv\left( {1 \times m_{i,j} \times k_{i,j} } \right)/func + Max\left( {1 \times p_{i,j} } \right), $$

where $func$ represents activation function and $Max\left( {1 \times p_{i,j} } \right)$ represents a max-pooling with region $1 \times p_{i,j}$.

Finally, the extracted single-scale features are reconstructed into an image of size $(l + 1) \times n$. Where n is the number of features after feature extraction for each scale.

**Figure 3:**
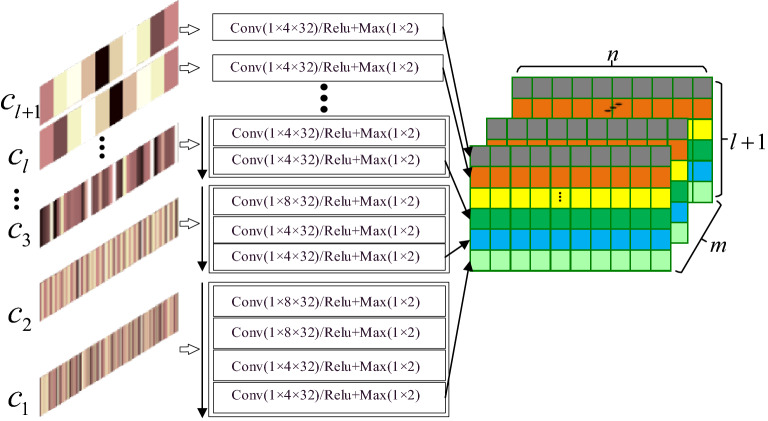
Single-scale feature extraction and recombination.

As shown in Fig. 3, 1D-CNN for $c_{1}$ can be illustrated with the scheme of

$$ \begin{gathered} c_{1} :ms \times 512 - \hfill \\ o_{1,1} : \, {\text{Conv}} \left( {1 \times {8} \times 32} \right)/{\text{Relu}} + {\text{Max}} \left( {1 \times 2} \right) - \hfill \\ o_{1,2} : \, {\text{Conv}} \left( {1 \times {8} \times 32} \right)/{\text{Relu}} + {\text{Max}} \left( {1 \times 2} \right) - \hfill \\ o_{1,3} : \, {\text{Conv}} \left( {1 \times {4} \times 32} \right)/{\text{Relu}} + {\text{Max}} \left( {1 \times 2} \right) - \hfill \\ o_{1,4} : \, {\text{Conv}} \left( {1 \times 4 \times 32} \right)/{\text{Relu}} + {\text{Max}} \left( {1 \times 2} \right). \hfill \\ \end{gathered} $$

It means a 1D-CNN with *ms* input sequences of size 1 × 512, a convolutional layer with 32 feature maps and 1 × 8 filters which is followed by a max-pooling with region 1 × 2, a convolutional layer with 32 feature maps and 1 × 8 filters which is followed by a max-pooling with region 1 × 2, a convolutional layer with 32 feature maps and 1 × 4 filters which is followed by a max-pooling with region 1 × 2, a convolutional layer with 32 feature maps and 1 × 4 filters which is followed by a max-pooling with region 1 × 2. The activation function of the convolution layer is Rectified Linear Units (ReLU). Once all single-scale features have been extracted, the extracted single-scale features are concatenated into a tensor of size $m \times (l + 1) \times n$. Where *m* is the channels of outputs and *n* is the number of features after feature extraction for each scale.

#### Multi-scale correlation feature extraction

Convolutional neural networks are very suitable for image-like data and can extract essential features because of its structure and convolution operation. After the single-scale feature extraction and recombination, 2D-CNN was adopted to extract correlation features of multi-scale information over the input sequence.

**Figure 4:**
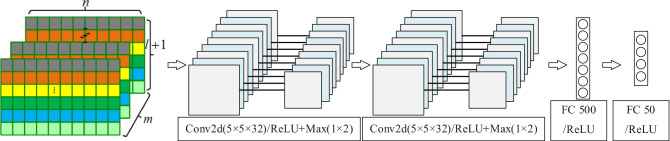
2D-CNN for multi-scale correlation feature extraction.

As shown in Fig. 4, the size of the input tensor is $m \times (l + 1) \times n$. The first convolutional layer takes as input the tensor and filters it with 32 convolutions of size 5 × 5 × *m*, which is followed by a max-pooling with region 1 × 2 to summarize the outputs. The second convolutional layer takes as input the (pooled) output of the first convolutional layer and filters it with 32 convolutions of size 5 × 5 × *m*, which is followed by a max-pooling with region 1 × 2. The latter two fully connected layers have 500 and 50 neurons respectively.

### Global time series dependency mining using multiple LSTM

LSTM is developed from RNN, inherits the recurrent structure of RNN and solves the problem of vanishing gradient or exploding gradient. It is good at dealing with complicated problems of long time series dependency. Therefore, LSTM is applied on local hybrid feature vectors to obtain long-term global dependencies. The hybrid features vector consists of automatic features generated by multilevel-CNN-based feature extraction model and manual features. The manual feature vector usually consists of time-domain, frequency-domain and time–frequency domain features^31^.

**Figure 5:**
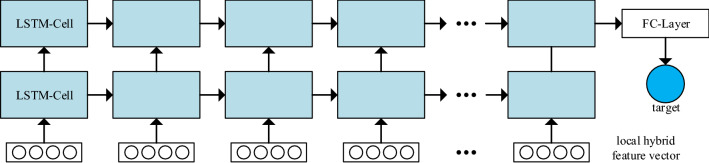
Global time series dependency mining using LSTM.

As shown in Fig. 5, the sequence of hybrid feature vectors is taken as input passing through multiple LSTM layers. The hidden output of an LSTM cell is not only passed to the next cell of the same layer over time, but also used as the input of next LSTM layer.

Figure 1 shows the basic architecture of the proposed local-feature and global-dependency based tool wear prediction model. The parameters of 2D-CNN extracting the feature of each time-series segment are shared, which are the same as 1D-CNNs. Therefore, the model parameters can be reduced and the essential features of tool wear in different time-series segments can be extracted. Moreover, the local hybrid features are input into the multi-layer LSTM in time series, which enables the model to mine the long-term dependency related to tool wear in the machining process.

## Case study and experimental results

In this section, an experiment was designed to test the performances of our proposed LFGD-TWP method.

### Introduction of experimental data

**Figure 6:**
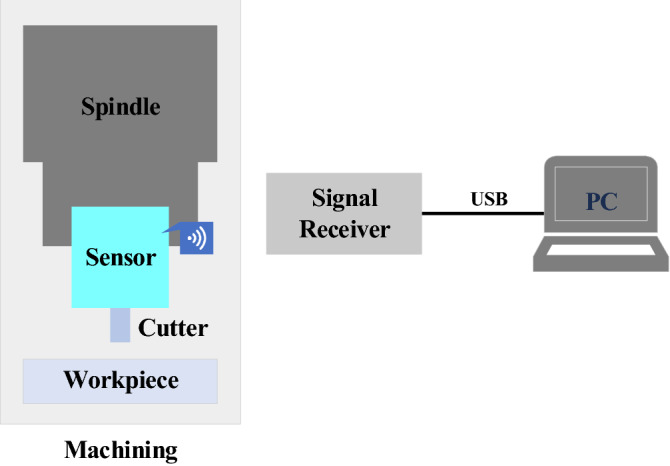
Signal acquisition.

The machining experiment was carried out in milling operation and the experimental equipment and materials used in this experiment are shown in Table 1. The cutting force acquisition system mainly consists of sensor, transmitter, receiver and PC. The sensor and signal transmitter are integrated into a toolholder, which can directly collect the force data during machining and send it out wirelessly. The signals are collected at a frequency of 2500 Hz. The collected data from sensor is transmitted wirelessly to receiver, which in turn transmits the data to PC via USB cable. The signal collection process is shown in Fig. 6.

**Table 1:** Experimental equipment and materials.

Hardware conditions	Model number
Experimental platform	SMU50 5-axis vertical machining center
Bending moment sensor	SPIKE
Torsion sensor	SPIKE
Wear measurement	Anyty Microscope 3R-MSUSB601
Milling cutter	Zhuzhou GM-4E-D10.0
Workpiece	1045

**Figure 7:**
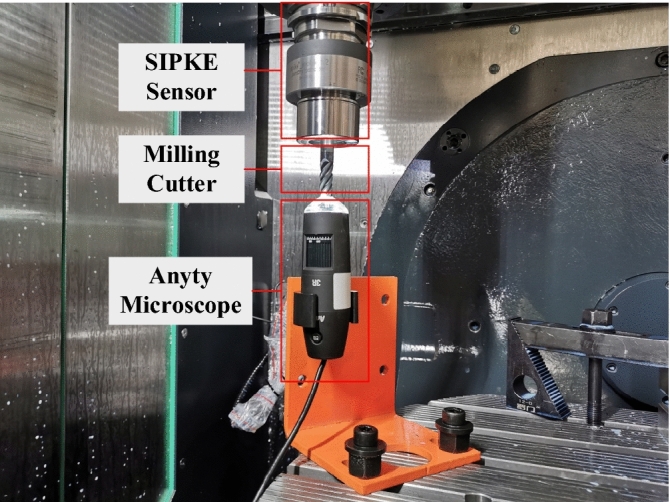
Tool wear measurement.

**Figure 8:**
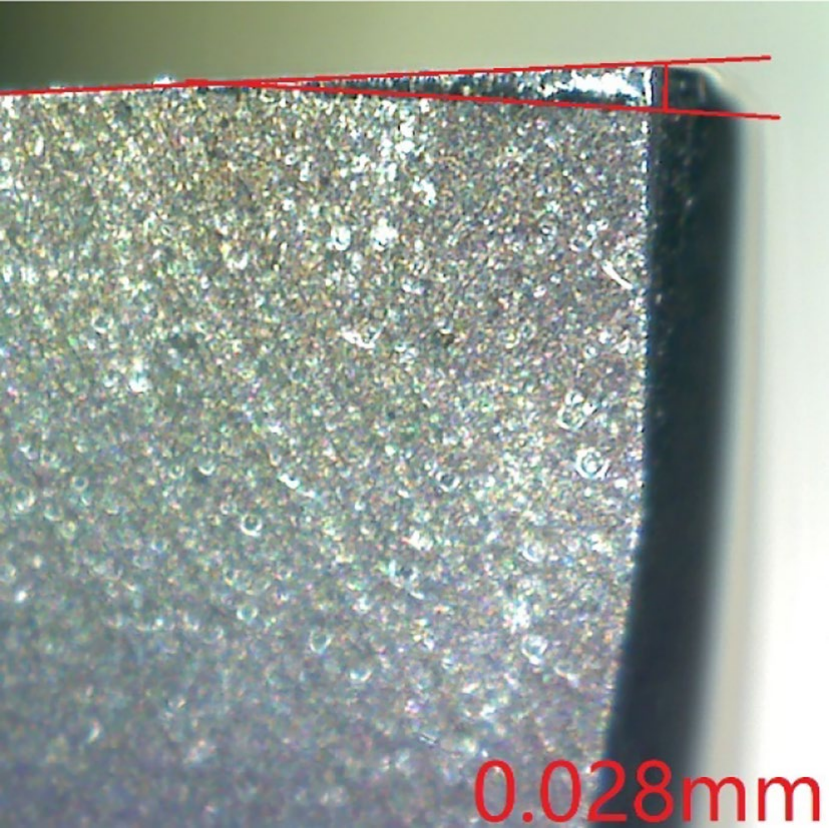
A sample photo of tool wear.

The Anyty microscope was fixed inside the machine tool as shown in Fig. 7. The coordinate where image of tool wear can be clearly taken is recorded into the CNC so that the spindle can move to this fixed position for wear measurement after each milling. This measurement method avoids the errors caused by repeated removal and installation of cutters, which improves the efficiency and accuracy of tool wear measurement. A sample photo of the microscope is shown in Fig. 8.

Orthogonal experimental method was adopted in this paper in order to test the performances of our method under multiple working conditions. Tool wear experiments are conducted using nine cutters under nine different cutting parameters. The 9 cutters are marked as C1, C2,…, C9. The milling parameters were set as shown in Table 2. The cutting width was fixed at 7 mm. Each row in the table corresponds to a new cutter. Every 1000 mm cutting was a cut and the tool wear was measured after every cut. Replace the cutter and cutting parameters when the previous tool wear exceeds the threshold or the cutter is broken.

**Table 2:** Parameter setting.

No.	Cutting speed (m/min)	Feed rate (mm/z)	Cutting depth (mm)
C1	150	0.1	0.2
C2	150	0.15	0.3
C3	150	0.2	0.4
C4	200	0.1	0.3
C5	200	0.15	0.4
C6	200	0.2	0.2
C7	250	0.1	0.4
C8	250	0.15	0.2
C9	250	0.2	0.3

The data acquisition files have three columns, corresponding to: bending moment in two directions (x, y) and torsion. Each cutter has a corresponding wear file. The wear file records the wear values of the four flutes corresponding to each cut. The cutting quality will become poor if the wear value of any edge exceeds a certain value. Therefore, this paper takes the maximal flank wear of all flutes as target.

### Results and discussion

#### Data preparation

Considering the multisensory input contain three channels, the bending moment in X direction is used as an example to illustrate the data preparation process in this paper. Firstly, the original signal of each cut is truncated to obtain the valid data segment containing 10,240 recorded values in the middle part of each signal. Finally, the data is equally divided into 10 segments based on practice, denoted as $X_{fx} = \left[ {X_{1} ,X_{2} ,...,X_{10} } \right]$.

#### Local time series data conversion

The maximum level of decomposition in DWT is related to the length of signals and the chosen wavelet. In this paper, db5 is used for decomposition and we select the optimal level of decomposition by comparing the performance under different levels of decomposition. Decomposition level 3, 4, 5 and 6 were chosen for comparison in this paper. The results showed that level 5 had the best performance. Therefore,$X_{1} ,X_{2} ,...,X_{10}$ are converted to multi-scale spectrogram images respectively by 5-level wavelet decomposition using db5 based on the practice, denoted as $WS = [ws_{1} ,ws_{2} ,...,ws_{10} ]$ where $ws = [c_{1} ,c_{2} ,...,c_{6} ]$ with the length of [512, 256, 128, 64, 32, 32] is multi-scal*e vectors corresponding to each segment.*

#### Local time series feature extraction

For each segment, 1D-CNNs are used to extract single-scale features from $c_{1} ,c_{2} ,...,c_{6}$ respectively. The structure and parameters of the model are shown in Table 3.

**Table 3:** Structure and parameters of single-scale feature extraction model.

$c_{1}$	$c_{2}$	$c_{3}$	$c_{4}$	$c_{5}$	$c_{6}$
Conv(1 × 8 × 32)	Conv(1 × 8 × 32)	Conv(1 × 4 × 32)	Conv(1 × 4 × 32)	Conv(1 × 4 × 32)	Conv(1 × 4 × 32)
Conv(1 × 8 × 32)	Conv(1 × 4 × 32)	Conv(1 × 4 × 32)			
Conv(1 × 4 × 32)	Conv(1 × 4 × 32)				
Conv(1 × 4 × 32)					

The activation function of the convolution layer is ReLU. Every convolution layer of $c_{1} ,c_{2} ,c_{3} ,c_{4}$ is followed by a max-pooling layer with region 1 × 2 to compress generated feature maps. The input channel of the model is set to 3 because of the three-channel sensory data.

After the single-scale Feature Extraction by 1D-CNNs and the concatenation of single-scale Features, a feature image of size ${32} \times {6} \times 32$ is obtained, which is used as the input of our multi-scale correlation feature extraction model. Finally, the local feature size of each segment after automatic extraction is 1 × 50.

#### Global time series dependency mining

In this case, the dimension of automatic feature vector is 50, and the dimension of manual feature vector is 30. The adopted manual features are shown in Table 4. Therefore, the dimension of the hybrid features of each segment is 80.

**Table 4:** Manual features for tool wear prediction.

Features	Equations
Mean	$\frac{1}{n}\sum\nolimits_{i = 1}^{n} {x_{i} }$
RMS	$Z = \sqrt {\frac{1}{n}\sum\nolimits_{i = 1}^{n} {x_{i}^{2} } }$
Variance	$\frac{1}{n}\sum\nolimits_{i = 1}^{n} {(x_{i} - \overline{x} )^{2} }$
Maximum	Max(x)
Minimum	Min(x)
Skewness	$E[(\frac{x - \mu }{\sigma })^{3} ]$
Kurtosis	$E[(\frac{x - \mu }{\sigma })^{4} ]$
Spectral skewness	$\sum\nolimits_{i = 1}^{k} {((f_{i} - \overline{f} )/\sigma )^{3} S} (f_{i} )$
Spectral Kurtosis	$\sum\nolimits_{i = 1}^{k} {((f_{i} - \overline{f} )/\sigma )^{4} S} (f_{i} )$
Spectral power	$\sum\nolimits_{i = 1}^{k} {(f_{i} )^{3} S} (f_{i} )$

The number of segments is T = 10 so that the shape of the input sequence of Global Time Series Dependency Mining Model is 80 × 10. The Mean Squared Error (MSE) was selected as the model loss during model training. An Adam optimizer^32^ is used for optimization in this paper and the learning rate is set to be 0.001. MSE was calculated on test data set for the models having one, two, and three layers and 100, 200, 300, 400, 500 hidden units. The results show that the most accurate model contained 2 layers and 300 hidden units in LSTM models and 400 hidden units in FC-Layer. In order to improve the training speed and alleviate the overfitting issues, we apply batch normalization (BN)^33^ to all convolution layers of Single-Scale Feature Extraction Model, and apply the dropout method^34^ to the fully connected layer. To get a relatively optimal dropout value, we set different values to train the model, i.e., p = 0, p = 0.25, p = 0.5, p = 0.75. Where p is the probability of an element to be zeroed. The results show that the dropout setting of 0.5 gives a relatively optimal result. After updating the parameters of the model with the training data, the trained model is applied on the testing data to predict tool wear.

In order to quantify the performance of our method, mean absolute error (MAE) and root mean squared error (RMSE) are adopted as measurement indicators to evaluate regression loss. The equations of MAE and RMSE over n testing records are given as follows:

5 $$ MAE = \frac{1}{n}\sum\limits_{i = 1}^{n} {\left| {y_{i} - \hat{y}_{i} } \right|} , $$

6 $$ RMSE = \sqrt {\frac{1}{n}\sum\limits_{i = 1}^{n} {(y_{i} - \hat{y}_{i} )^{2} } } , $$

where $y_{i}$ is predicted value and $\hat{y}_{i}$ is true value.

To analyze the performance of all our methods, cross validation is used to test the accuracy of the model in this paper. Eight cutter records are used as training sets and the rest one is used as testing set, until all cutters are used as testing set. For example, records of cutters C2, C3, …, C9 are used as the training sets and records of cutter C1 are used as the testing set, the testing case is denoted as T1. Then the records of cutter C2 are used as the testing set, and the records of the rest cutter are used as the training sets, the testing case is denoted as T2. The rest can be done in the same manner. Nine different testing cases are shown in Table 5.

**Table 5:** Testing cases.

Testing cases	Training	Testing
T1	C2, C3, C4, C5, C6, C7, C8, C9	C1
T2	C1, C3, C4, C5, C6, C7, C8, C9	C2
T3	C1, C2, C4, C5, C6, C7, C8, C9	C3
T4	C1, C2, C3, C5, C6, C7, C8, C9	C4
T5	C1, C2, C3, C4, C6, C7, C8, C9	C5
T6	C1, C2, C3, C4, C5, C7, C8, C9	C6
T7	C1, C2, C3, C4, C5, C6, C8, C9	C7
T8	C1, C2, C3, C4, C5, C6, C7, C9	C8
T9	C1, C2, C3, C4, C5, C6, C8, C8	C9

To mitigate the effects of random factors, each testing case is repeated 10 times and the average value is used as the result of the model. Moreover, in order to demonstrate the effectiveness of the hybrid features in this paper, two models are trained, namely the network with hybrid features and the network with automatic features only. The results of each testing cases are shown in Table 6.

**Table 6:** Measurement indicator (MAE and RMSE) achieved by LFGD-TWP.

Testing cases	Hybrid feature		Automatic features		Performance improvement	
	MAE	RMSE	MAE	RMSE	MAE (%)	RMSE (%)
T1	5.52	7.03	5.68	7.64	2.82	7.98
T2	7.82	10.34	8.04	10.29	14.07	11.47
T3	9.43	12.15	11.25	15.14	16.18	19.75
T4	7.73	10.08	9.1	11.68	3.86	2.04
T5	7.41	9.86	6.72	8.49	− 10.27	− 16.14
T6	6.81	8.76	6.92	9.14	1.59	4.16
T7	7.40	9.79	7.15	8.95	− 3.50	− 9.39
T8	7.08	9.59	7.16	9.44	1.12	− 1.59
T9	7.03	9.21	7.59	9.5	7.38	3.05

**Figure 9:**
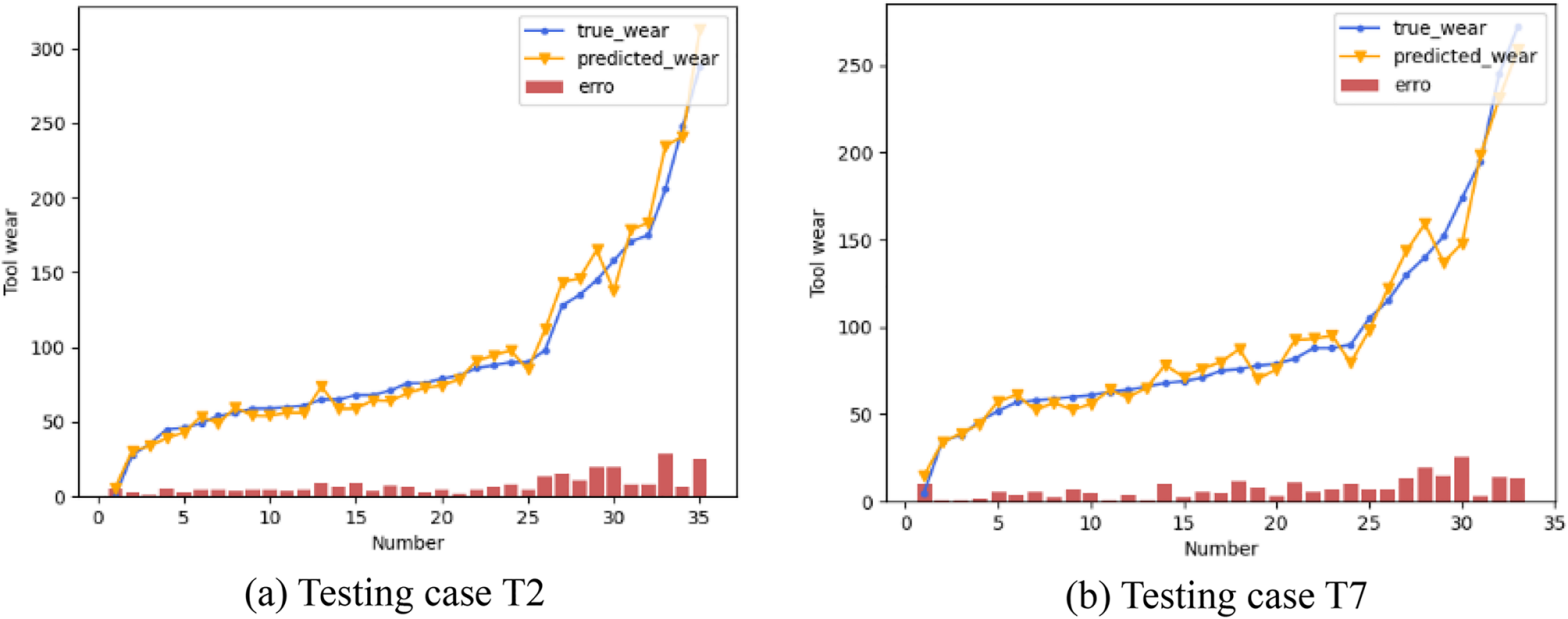
Tool wear predicted by LFGD-TWP.

It can be seen from Table 6 that our proposed LFGD-TWP achieves low regression error. In most cases, the model with hybrid features performs better than the model with automatic features only. By calculating the average performance improvement, we can reach a 3.69% improvement in MAE and a 2.37% improvement in RMSE. To qualitatively demonstrate the effectiveness of our model, the predicted tool wears of testing case T2 and T7 are illustrated in Fig. 9. It can be seen from Fig. 9 that the closer to the tool failure zone, the greater the error. The reason for this may be that the tool wears quicker at this stage, resulting in a relatively small number of samples. Or it could be that the signal changes more drastically and the noise is more severe due to the increasing tool wear, leading to greater error.

Two statistics are adopted to illustrate the overall prediction performance and generalization ability of the model under different testing cases: mean and variance. Mean is the average value of the results under different testing cases. Obviously, it indicates the prediction accuracy of the method. Variance measures how far each result is from the mean and thus measures variability from the average or mean. It indicates the stability of generalization under different testing cases. The equations of mean and variance of two measurement indicators over n testing cases are given as follows:

7 $$ Mean = \overline{r} = \frac{1}{n}\sum\limits_{i = 1}^{n} {r_{i} } , $$

8 $$ Variance = \frac{1}{n}\sum\limits_{i = 1}^{n} {\left( {r_{i} - \overline{r}} \right)^{2} } , $$

where $r_{i}$ is the mean value of the results for each testing case.

The definition of mean and variance shows that the smaller their values are, the better performance of the model will be. In our proposed method, the means of MAEs and RMSEs are 7.36 and 9.65, and the variances of MAEs and RMSEs are 0.95 and 1.65.

### Further comparison

Other deep learning models are used to compare model performance with the proposed LFGD-TWP. They are CNN^24^, and LSTM^30^ and CNN-BiLSTM^19^, and the structure of these models are shown as follows.

Structure of CNN model in brief: The input of CNN model is the original signal after normalization, and the signal length is 1024. The input channel of the model is set to 3 because of the three-channel sensory data. CNN model has 5 convolution layers. Each convolutional layer has 32 feature maps and 1 × 4 filters which is followed by a max-pooling with region 1 × 2. Then flatten the feature maps. Finally, it is followed by a fully connected layer, which has 250 hidden layer units. The dropout operation with probability 0.5 is applied to the fully connected layer. The loss function is MSE, the optimizer function is Adam, the learning rate is set to be 0.001, which are kept the same as the proposed model. The means of MAEs and RMSEs are 12.64 and 16.74, and the variances of MAEs and RMSEs are 10.74 and 18.90.

Structure of LSTM model in brief: The model is of type many to one. The input of LSTM is the manual features in Table 4. Therefore, an LSTM cell has an input dimension of 30. The MAE and RMSE values were calculated for models with one, two, and three layers and 100, 200, 300, 400 hidden units. Therefore, 12 structures of an LSTM model were constructed for the most accurate model. Also, the timesteps are 10, the loss function is MSE, the optimizer function is Adam, the learning rate is set to be 0.001, which are kept the same as the proposed model. The results show that the most accurate model contained 2 layers and 200 hidden units. The means of MAEs and RMSEs are 10.48 and 13.76, and the variances of MAEs and RMSEs are 5.12 and 9.28.

Structure of CNN-BiLSTM model is shown in Ref.^19^, and the input of this model is the original signal after normalization. The means of MAEs and RMSEs of this model are 7.85 and 10.24, and the variances of MAEs and RMSEs are 2.71 and 5.06. Comparison results of our method (LFGD-TWP) and popular models are shown in Table 7. Compared to the most competitive result achieved by CNN-BiLSTM, the proposed model achieves a better accuracy owing to the multi-frequency-band analysis structure. Further, it can be seen that the proposed model achieves lower variances in MAE and RMSE. It means that the proposed model has better overall prediction performance and better stability of generalization under different testing cases by comparing the variance of the results.

**Table 7:** Comparison results.

Algorithms	MAE		RMSE	
	Mean	Variance	Mean	Variance
CNN	12.64	10.74	16.74	18.90
LSTM	10.48	5.12	13.76	9.28
CNN-BiLSTM	7.85	2.71	10.24	5.06
LFGD-TWP	7.36	0.95	9.65	1.65

**Figure 10:**
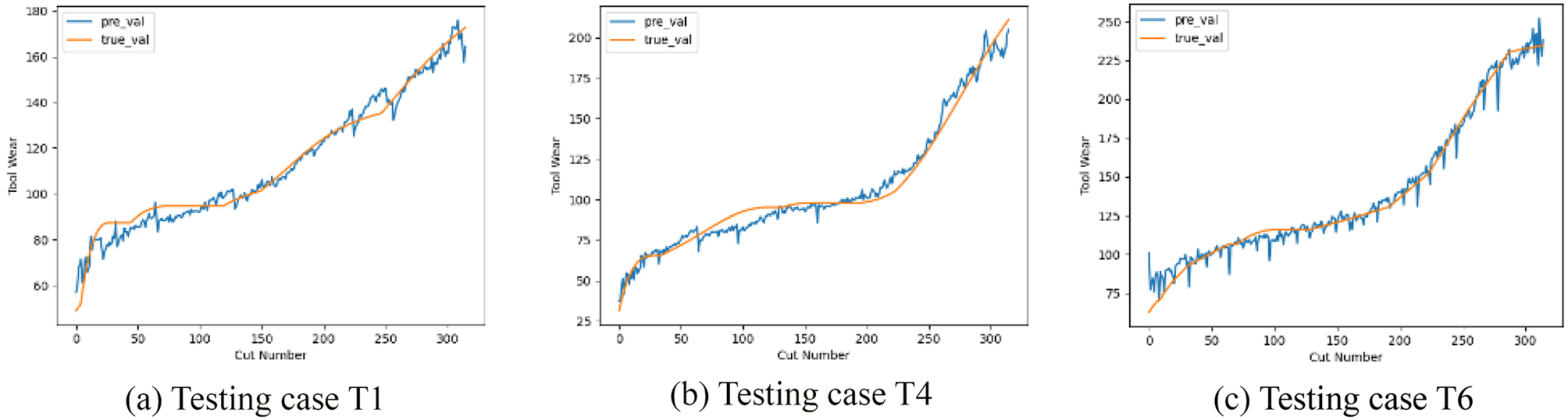
Tool wear (PHM2010) predicted by LFGD-TWP.

To further test the performance of our proposed method, we additionally use the PHM2010 data set^35^, which is a widely used benchmark. The machining experiment was carried out in milling operation and the experimental equipment and materials used in this experiment are shown in Ref.^19^. The running speed of the spindle is 10,400 r/min; the feed rate in x-direction is 1555 mm/min; the depth of cut (radial) in y-direction is 0.125 mm; the depth of cut (axial) in z-direction is 0.2 mm. There are 6 individual cutter records named C1, C2,…, C6. Each record contains 315 samples (corresponding to 315 cuts), and the working conditions remain unchanged. C1, C4, C6 each has a corresponding wear file. Therefore, C1, C4, C6 are selected as our training/testing dataset. Also, cross validation is used to test the accuracy of the model and the results are shown in Fig. 10.

In our proposed method, the mean of MAEs is 6.65, the mean of RMSEs is 8.42. Compared with the mean value of MAEs (6.57) and RMSEs (8.1) in Ref.^19^. The reason for the slightly poor performance may be that in order to enhance the adaptability to multiple working conditions, the architecture of the model is more complex, which leads to overfitting. Although the proposed architecture might overfit the PHM2010 case, the complexity of the architecture ensures that more complex scenarios like the test cases in the paper can be handled.

## Conclusions

In this paper, a new tool wear prediction method LFGD-TWP based on deep learning has been proposed. It combines manual feature extraction with automatic feature extraction based on machine learning. Enhanced CNN is used to extract the local features of a time series segment. Single-scale features are extracted from the wavelet transformed data by different depths respectively, and finally the single-scale features are combined for multi-scale feature extraction. Both the specific extraction of different single-scale features and the correlation extraction of multi-scale features are achieved. And multiple LSTM is used to extract the long-term dependency of multiple time series segments. Mean and variance are adopted to illustrate the overall prediction performance and generalization ability of the model under different testing cases. Good results are obtained in predicting tool wear under multiple working conditions.

However, the method proposed in this paper still has limitations. The sample frequency of this method is 2500 Hz, and we still need to do some extra work when processing data at different sample frequencies. Generally speaking, the actual level of decomposition depends on specific application scenarios. The features of tool wear exist in different frequency bands, and the change of sample frequency may affect the feature expression of tool wear. Therefore, the optimal level of decomposition may vary with sample frequency. When the sample frequency is higher than that in this paper, we can keep the sample frequency consistent with that in this paper by downsampling. Therefore, the architecture of the model will not be changed. When the sample frequency is lower than that in this paper, we can choose the level of decomposition by comparing the performance of different levels, which is the same as that in this paper. In the future work, we are going to explore the performance of our framework in a wider range of scenarios to improve the applicability of our model in intelligent manufacturing systems. For example, tool wear prediction under variable working conditions and tool wear prediction with variable sample frequency.

## Data Availability

The datasets generated and/or analysed during the current study are not publicly available due the data also forms part of an ongoing study but are available from the corresponding author on reasonable request.
